# Site‐specific metastasis: A cooperation between cancer cells and the metastatic microenvironment

**DOI:** 10.1002/ijc.33247

**Published:** 2020-08-27

**Authors:** Sivan Izraely, Isaac P. Witz

**Affiliations:** ^1^ The Shmunis School of Biomedicine and Cancer Research, George S. Wise Faculty of Life Sciences Tel‐Aviv University Tel Aviv Israel

**Keywords:** brain metastasis, cancer, metastatic microenvironment, site‐specific metastasis

## Abstract

The conclusion derived from the information provided in this review is that disseminating tumor cells (DTC) collaborate with the microenvironment of a future metastatic organ site in the establishment of organ‐specific metastasis. We review the basic principles of site‐specific metastasis and the contribution of the cross talk between DTC and the microenvironment of metastatic sites (metastatic microenvironment [MME]) to the establishment of the organ‐specific premetastatic niche; the targeted migration of DTC to the endothelium of the future organ‐specific metastasis; the transmigration of DTC to this site and the seeding and colonization of DTC in their future MME. We also discuss the role played by DTC‐MME interactions on tumor dormancy and on the differential response of tumor cells residing in different MMEs to antitumor therapy. Finally, we summarize some studies dealing with the effects of the MME on a unique site‐specific metastasis—brain metastasis.

**Abbreviations**
ANGPTL4: angiopoietin‐like 4
BBB: blood‐brain barrier
BMP: bone morphogenetic protein
CAM: cell adhesion molecules
CLDN1: claudin‐1
CysC: cystatin C
DTC: disseminating tumor cells
EC: endothelial cells
EMT: epithelial‐to‐mesenchymal transition
EV: extracellular vesicles
HBB: beta‐subunit of human hemoglobin
L1CAM: L1 cell adhesion molecule
MME: metastatic microenvironment
PMME: premetastatic microenvironment
TRPA1: transient receptor potential ankyrin 1
VCAM‐1: vascular cell adhesion molecule 1
XIST: X‐inactive specific transcript

## 1 INTRODUCTION

Stephen Paget is the conceptual father of the concept that metastasis is site‐specific. In his description of the “seed and soil” theory^1^ he wrote: “When a plant goes to seed, its seeds are carried in all directions; but they can only live and grow if they fall on congenial soil”. In contemporary language, the seeds are metastasizing cancer cells and the soil is the microenvironment of the organ hosting these cells.

The major research thrust on site‐specific metastasis is directed toward several targets: defining the molecular signature of cancer cells that enable their establishment as metastatic lesions in different organs; the molecular signature of the host microenvironmental cells supporting or inhibiting this establishment; the modus operandi by which such molecules exert their pro‐ or antimetastasis functions; the functional significance of interactions between metastasizing cancer cells with cells residing in or recruited to the microenvironment of specific organ sites and the impact of site‐specific metastasis on and response to therapy. The following references encompass a partial list of recent reviews on cancer metastasis that alluded to organ specificity.^2^, ^3^, ^4^, ^5^, ^6^, ^7^, ^8^, ^9^, ^10^, ^11^, ^12^, ^13^, ^14^

This review will summarize the principles governing organ‐specific metastasis and focus on interactions between cellular and molecular components of the metastatic microenvironment (MME) of specific organs with cancer cells infiltrating into the microenvironment of these organs.

The microenvironment of an organ belonging to a tumor bearer without evidence of metastasis in this organ will be designated as premetastatic microenvironment (PMME). The microenvironment of the same organ harboring micro‐ or macrometastasis will be designated as MME.

## 2 REVISITING SITE‐SPECIFIC METASTASIS IN THE POST‐PAGET ERA

It is well established that tumor cells interact by multiple mechanisms with cellular and molecular components of their microenvironment. These interactions reprogram and shape the phenotype of both interaction partners. In certain cases, such interactions may lead to metastasis.^15^, ^16^, ^17^, ^18^, ^19^, ^20^, ^21^, ^22^, ^23^, ^24^, ^25^, ^26^, ^27^, ^28^, ^29^

The concept of site‐specific metastasis lay dormant until the 1980s. The awakening of this concept revealed the basic principles of site‐specific metastasis as we know them today.^30^, ^31^, ^32^, ^33^, ^34^, ^35^

1. Different cancer types usually metastasize to multiple but favored organ sites. Breast cancer for example, metastasizes to bone, lungs, liver and brain while prostate cancer metastasizes primarily to lymph nodes and to bones.^36^ Since the microenvironment of different organs is different and in view of the fact that the tumor‐microenvironment interactions shape the phenotype of both interaction partners,^19^ it is to be expected that metastases derived from a single tumor of a single patient but developing in different organs, be different.
2. The major determinants of site‐specific metastasis are alterations in the genetic, epigenetic and proteomic profiles of metastasizing cancer cells as well as factors derived from the MME.^32^, ^37^, ^38^
3. Although tumor cells reach the vasculature of all organs, metastasis develops only in selected organs. Specific adhesive recognition between cancer cells and the endothelium of the target organ, as well as nonspecific anatomical or mechanical factors such as circulation mechanics are involved in the arrest of circulating cancer cells at the target organ.^5^, ^37^, ^39^, ^40^, ^41^, ^42^, ^43^
4. The ability of metastasizing tumor cells to survive and propagate at a specific organ site, being an essential prerequisite for metastasis formation, is enabled to a large extent by MME‐derived survival and growth factors.^44^, ^45^, ^46^

With these issues in mind, it is essential to be aware of the possibility that many, perhaps most, events related to tumor progression in general, occur simultaneously and that most such events may yield Yin‐Yang results.^19^, ^20^

This review will focus on the involvement of the MME in the four major phases of site‐specific metastasis, namely creation of the premetastatic niche; migration of metastasizing tumor cells to specific organ sites; invasion of the tumor cells into the target organ and the establishment of dormant or overt metastasis in this organ.

## 3 PMME PLAYS A DOMINANT ROLE IN PREMETASTATIC NICHE FORMATION

Kaplan and coworkers were the first to demonstrate that future metastatic sites are preconditioned for colonization of circulating tumor cells.^47^ The formation of the premetastatic niche and its activities depend totally on an active cross talk between three partners: tumor cells, bone‐marrow derived cells and the PMME.^11^, ^48^

The initial step of premetastatic niche formation in specific organ sites is mediated by tumor‐derived soluble factors such as TNF‐α, TGF‐β, CXCL12, placental growth factor and VEGF‐A^49^ as well as by tumor‐derived exosomes.^50^, ^51^, ^52^ These factors are responsible for the recruitment of bone marrow‐derived hematopoietic progenitor cells via the upregulation of specific molecules such as fibronectin, S100A8, S100A9, MMP2, MMP9 and LOX in the PMME. These PMME molecules communicate with the distant bone marrow, thereby recruiting hematopoietic progenitor cells (CD34+, VEGFR1+) and myeloid cells (CD11b+).^53^, ^54^

**FIGURE 1:**
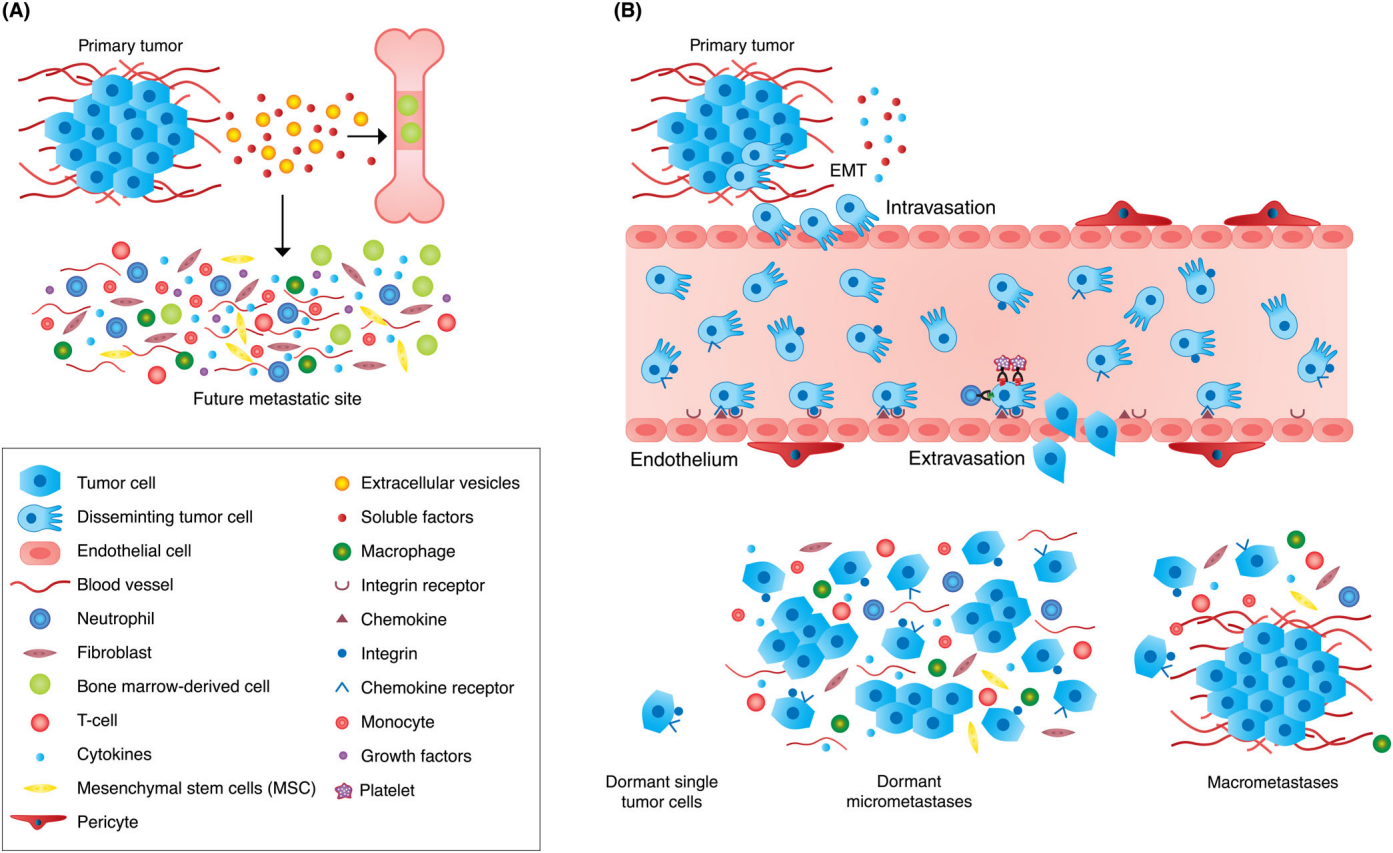
Site‐specific metastasis and the metastatic microenvironment (MME). A, Formation of the premetastatic microenvironment. Extracellular vesicles (EVs) (including exosomes) as well as soluble factors released from primary tumor cells induce the release of progenitor cells from bone marrow and their targeted migration to a specific future metastatic site. The tumor‐derived EVs and soluble factors interact also with this specific future metastatic site. Both the bone marrow‐derived cells as well as the tumor‐derived EVs and soluble factors play crucial roles in conditioning such premetastatic niches for future metastatic colonization.^48^, ^49^, ^50^, ^51^, ^52^, ^53^, ^54^, ^55^, ^56^ The premetastatic niche is generated by soluble factors (eg, cytokines and growth factors), EVs, stromal cells and regulatory or suppressive immune cells, including mesenchymal stem cells (MSCs), fibroblasts, T‐cells, macrophages, neutrophils and monocytes that can promote tumor cell colonization and metastasis.^256^ B, Disseminating tumor cells (DTC)—relocation from primary to secondary sites; extravasation and colonization. Tumor cells that acquire a mesenchymal phenotype by employing the epithelial‐to‐mesenchymal transition—(EMT) mechanism,^18^, ^61^, ^62^, ^63^, ^64^, ^65^, ^66^ detach from the primary tumor, find their way to the circulation and intravasate. Using chemokine‐chemokine receptor axes^70^, ^71^, ^72^, ^73^, ^74^, ^75^, ^76^, ^77^, ^78^, ^79^, ^80^ and interaction with distant organ blood vessel components, including pericytes and site‐specific endothelial adhesion molecules (such as integrins),^31^, ^84^, ^106^, ^107^, ^218^ the circulating tumor cells extravasate into existing premetastatic niches thereby creating the MME. Aggregation of DTC with neutrophils or with platelets also endorses adhesion and extravasation of the endothelium^106^, ^110^, ^111^ thereby promoting metastasis. Under MME influence, the incoming DTC either do not survive; remain as dormant solitary cells or micrometastases,^174^, ^178^, ^179^, ^180^, ^181^, ^182^, ^184^ or progress toward macrometastasis^190^, ^191^, ^192^, ^193^

The recruited cells play very important functions in creating, in a foreign milieu, a hospitable microenvironment for incoming tumor cells and for their progression toward metastasis^49^, ^55^ (Figure 1A).

The cargo of exosomes contains single‐ or double‐stranded DNA, mRNA and noncoding RNAs, proteins and various types of glycans. All these molecules, directly or indirectly exert numerous and varied effects on the PMME, MME and tumor cells alike. For example, exosomes increase vascular leakiness, reprogram the ECM, suppress anti‐tumor immune reactivity and promote tumor progression.^56^

One of the most important and relevant issues regarding premetastatic niche formation (and to the topic of this review) is the mechanism by which tumor‐derived exosomes locate the “correct address” of the organ, which will serve as the future home for metastasis originating in the corresponding tumor. In other words, what are the specific recognition units expressed on the surface of exosomes that mediate the interaction with the target organ via complementary ligands and what are these ligands?

Several molecular mechanisms may underlie the organotropism of tumor‐derived exosomes (and the subsequent formation of metastasis specific to a particular organ).^57^, ^58^, ^59^

The most extensive analysis of the organotropism of tumor‐derived exosomes was performed by Hoshino and collaborators^60^ who identified integrins as the molecules directing exosomes to the organ, which will house future metastases. For example, exosomes derived from a breast carcinoma cell line expressing α6β4 and α6β1 integrins, localized, in vivo, in lung regions expressing high levels of laminin, a ligand for these integrins. A similar observation was reported regarding a pancreatic cancer cell line, which preferentially produces liver metastasis. Exosomes derived from this cell line expressed αvβ5 and localized in regions of the liver rich in fibronectin, which serves as a ligand for αvβ5.

In view of the above, it is not unlikely that integrin‐integrin ligand axes are the major mechanisms responsible for the organotropism of tumor‐derived exosomes and for site‐specific metastasis. In order to prepare tailor‐made therapy modalities for metastasis lodging in different organs, it is essential to fully assess the unique molecular and pathological processes that may occur in each MME before the development of metastases.

## 4 THE RELOCATION OF TUMOR CELLS FROM PRIMARY TO SECONDARY SITES

Tumor cells disseminating from the primary tumor (disseminating tumor cells—DTC) reach secondary organ sites by employing two major, nonmutually exclusive mechanisms. The one, triggered by the TME, involves the reprograming of adhesive epithelial cancer cells to migratory and invasive mesenchymal cells (epithelial‐to‐mesenchymal transition—EMT). This general mechanism may culminate in metastasis formation.^18^, ^61^, ^62^, ^63^, ^64^, ^65^, ^66^

The machinery of targeted migration employed by inflammatory leukocytes to reach inflammatory sites^67^, ^68^, ^69^ is hijacked by DTC to reach future metastatic sites. Inflammatory chemokines released from cells residing in the microenvironment of future metastatic sites interact with chemokine receptors expressed by DTC, thereby enabling their targeted migration to such sites^70^, ^71^, ^72^, ^73^ (Figure 1B). Among the chemokine‐chemokine receptor axes enabling targeted migration of tumor cells to specific organ sites are CXCR4‐CXCL12,^74^ CCR7‐CCL21,^75^ CCR6‐CCL20,^76^ CXCR1 and CXCR2‐CXCL8^77^ and others.^72^

A study from our laboratory established that the chemokine receptor CCR4 is a member of the molecular signature of melanoma brain metastasis and that its chemokine ligands CCL17 and CCL22 are expressed in microenvironmental brain cells.^78^ These findings raised the possibility that the CCR4‐CCL17/CCL22 axis takes part in the targeted migration of melanoma cells to the brain as well as in the formation of metastasis in this organ. This was indeed the case. Cross talk between melanoma cells and microglia, astrocytes and brain endothelial cells (EC) upregulated CCR4 expression on melanoma cells and CCL17/CCL22 expression by and secretion from the brain cells. CCR4 overexpressing melanoma cells were more highly tumorigenic and generated more brain micrometastases than control cells. A small molecule antagonist of the CCR4‐CCL17/CCL22 axis reduced the malignancy of melanoma cells.^79^, ^80^

In addition to directing tumor cells to specific organ sites via “cellular highways,” chemokine‐chemokine receptor axes exert other metastasis‐facilitation functions such as promotion of tumor proliferation and of angiogenesis as well as recruitment of suppressor immunocytes.^73^

## 5 TUMOR‐BLOOD VESSEL INTERACTIONS AND EXTRAVASATION OF DISSEMINATED TUMOR CELLS

### 5.1 General features

In addition to the entrapment of DTC in small capillaries of most organs,^43^ specific interactions between DTC and blood vessels (pericytes, endothelium, subendothelium) are also recognized as a major factor in site‐specific dissemination (see later).^81^

Pericytes are involved in tumor progression toward metastasis.^82^ One mechanism by which pericytes exert their prometastatic function is by relocation to the MME and their transition to cancer‐associated fibroblasts (see later). This, in turn, supports tumor progression toward metastasis.^83^, ^84^ Pericytes, similarly to other TME/MME components, may exert Yin‐Yang functions with respect to tumor progression. For example, brain pericytes may function to maintain the integrity of the blood‐brain barrier (BBB), thereby suppressing the formation of lung cancer metastasis.^85^

ECs of one particular organ often express a different profile of cell surface proteins compared to ECs of other organs. This organ specificity accounts for the fact that DTC bind specifically to the vasculature of some organs but not to that of others.^86^, ^87^, ^88^, ^89^, ^90^, ^91^, ^92^ DTC‐endothelial interactions regulate the extravasation of the tumor cells and subsequently take part in determining organ preferences of metastatic spread.^31^, ^88^, ^93^, ^94^, ^95^

Leukocyte extravasation is a multistep process whereby leukocytes expressing fucosylated selectin ligands role over cytokine‐activated selectin‐expressing ECs. Subsequently the leukocytes encounter chemokines expressed on the surface of ECs. This encounter triggers the activation of leukocyte‐expressed integrins and subsequently a firm adhesion to the activated endothelium. Eventually, diapedesis regulated by the activation of Rho GTPases takes place.^96^, ^97^

The complex extravasation cascade of leukocytes during inflammatory responses was co‐opted (some would use the term hijacked) by circulating cancer cells. This co‐option is manifested inter alia by the utilization of surface molecules involved in leukocyte extravasation also for endothelial transmigration of DTC.^98^, ^99^, ^100^, ^101^, ^102^, ^103^, ^104^, ^105^, ^106^

Several cell surface molecules including integrins, selectins, other cell adhesion molecules, glycosyl transferases, glycolipids and fetal recognition systems^31^, ^100^, ^102^, ^107^, ^108^ mediate DTC‐EC adhesive interactions. Chemokines, exosomes secreted from tumor or stroma cells as well as local inflammatory/immune responses at the target organ contribute to the extravasation process^58^, ^105^, ^109^ (Figure 1B).

The co‐aggregation of DTC with neutrophils^110^ or with platelets^106^, ^111^ also promotes adhesion to the endothelium, extravasation and subsequently capacity to metastasize.

### 5.2 Site‐specific interaction between tumor and ECs

Early studies demonstrating the organ‐specific nature of DTC‐EC binding were performed by Auerbach et al.^86^ They showed that capillary ECs express on their cell surface an array of organ‐specific antigens. Brain‐derived ECs, for example, express brain‐associated antigens, ovary‐derived ECs express ovary‐associated antigens and lung‐derived ECs express lung‐associated antigens. Some of these EC recognition molecules are expressed constitutively, and others are induced by environmental signals, such as cytokines or free radicals.^95^ Auerbach et al also provided the first experimental evidence that organ‐specific metastasis is determined by a selective adherence of tumor cells to organ‐associated determinants on EC surfaces.^112^ Analyzing the adhesion of various tumor types to ECs derived from different organs indicated that tumor cells differ in their adhesive preference for different ECs, usually in correlation to the pattern of metastatic spread of these tumors.

Several adhesion molecules expressed by DTC or by endothelia of specific organs are determinants of site‐specific metastasis. Molecular determinants and mechanisms mediating adhesion of DTC to EC and transendothelial migration were characterized with respect to lymph nodes,^106^, ^107^ liver,^58^, ^113^, ^114^, ^115^, ^116^, ^117^ bone,^58^, ^118^, ^119^ lung,^58^, ^106^, ^109^, ^117^, ^120^, ^121^, ^122^, ^123^, ^124^, ^125^, ^126^ kidneys^117^ and brain.^58^, ^125^, ^126^, ^127^, ^128^, ^129^, ^130^ Below are examples of signaling pathways involved in the organ‐specific interaction between cancer cells and endothelium: colonization of E‐selectin ligands‐expressing liver metastasizing cells requires the expression of E‐selectin by liver EC. Lung‐metastasizing tumor cells need the expression of the lung vasculature adhesion molecule, LuECAM to colonize lungs.^101^ Melanomas and Lewis lung carcinomas upregulate angiopoietin‐2 (ANG2) expression selectively in the lungs. Secreted ANG2 facilitates the expression of EC adhesion molecule as well as EC junction disruption (via the formation of VE‐cadherin‐TIE2‐ANG1 complexes) leading to lung metastasis.^109^ The β3 integrin heterodimers α2bβ3 and αvβ3 are required for the adhesion and aggregation of melanoma cells to platelets, important for arresting melanoma cells in the bone capillaries.^106^ The CD44 protein mediates the adhesion of myeloma, breast and prostate cancer cells to bone marrow ECs, eventually leading to their extravasation.^105^ The α4β1 integrin expressed by melanoma cells binds vascular cell adhesion molecule 1 (VCAM‐1) expressed by lymph node ECs. The same proteins, each expressed by the counter cell (α4β1 integrin by ECs and VCAM‐1 by melanoma cells), also play a functional role in melanoma adhesion to lymph node ECs.^106^

## 6 SEEDING AND COLONIZATION

### 6.1 General aspects

Disseminated tumor cells endowed with a genetic repertoire that sanctions their colonization in the PMME of a specific organ^36^, ^127^, ^131^, ^132^, ^133^, ^134^ are subject to evolutionary steps dictated by the specific PMME of the host organ. The evolution of metastasizing tumor cells in the MME may culminate either in successful establishment of a metastatic lesion, in dormancy/quiescence or in demise of such cells.

A case in point is the production of reactive oxygen and nitrogen species either by DTC themselves or by the PMME/MME. The exposure of the metastasizing cancer cells to the oxygen/nitrogen‐mediated cytotoxic insults may lead either to the survival of highly resistant tumor variants and/or to the elimination of sensitive ones.^135^

In general, ECM, the vascular system, immunity, metabolism and other cellular and molecular constituents of the PMME/MME engage in a cross talk with DTC leading to their colonization in various organ sites.^136^

In the following, there are several examples for tumor‐promoting mechanisms that are not selective for specific DTC/MME combinations.

The contribution of fibroblasts residing in malignant tumors (cancer‐associated fibroblasts—CAFs) to cancer progression toward metastasis has been thoroughly studied.^137^, ^138^ The origin and functions of metastasis‐associated fibroblasts (MAFs), and their relationship with CAFs are, however, less well known and require further examination.^139^ CAFs from human breast cancer tissues were transformed, in vitro to MAFs when cocultured with tumor cells carrying a mutated BRCA1 gene, suggesting that mutational events in cancer cells could generate reprograming in interacting fibroblasts.^140^ MAFs promoted the malignancy of pancreatic cancer metastasis inducing angiogenesis^141^ or promoted the propagation of lung‐metastasizing murine mammary carcinoma cells.^142^

Hepatic stellate cells are transformed by colorectal cancer‐derived signals to fibroblasts. These hepatic fibroblasts express CCL12, thereby contributing to the targeted migration of CXCR4‐expressing cancer cells to the liver.^143^

Macrophages in the premetastatic niche promote tumor invasion into the niche and subsequently take part in the generation of metastasis. Macrophages within tumor/metastatic lesions (tumor‐associated macrophages—TAMs) promote propagation of tumor cells, induce matrix remodeling and angiogenesis, and establish an immunosuppressive microenvironment^144^ and as such fulfill a major role in colonization of cancer cells in secondary organs.

Neurogenesis is a determinant in the seeding/colonization of tumor cells in the MME.^145^ Neurogenesis plays a role in prostate tumors that are infiltrated by neural progenitors from the central nervous system. These progenitors promote metastasis.^146^ MicroRNAs (miRNAs) link neurogenesis with metastasis. The miRNAs function as bothregulators of neurogenesis as well as of the metastatic potential of tumor cells.^147^, ^148^

The L1 cell adhesion molecule (L1CAM) is used by DTC to spread on capillaries of the PMME/MME. The spreading leads to activation of Yes‐associated protein (YAP) being a necessary component of colonization of various types of cancer in different organs.^149^

Essentially all cancer types evoke inflammatory reactions and antitumor immunity at the microenvironment of the primary tumor as well as in the MME of essentially all organ sites. These responses may either promote or restrain tumor progression.^150^, ^151^, ^152^, ^153^, ^154^, ^155^ Primary tumor or metastatic cells may escape from immune insults by a variety of general mechanisms such as immune suppression, loss or downregulation of MHC‐I or tumor associated antigens, hijacking immune regulatory mechanisms such as recruitment of suppressor cells or immune checkpoint pathways.^156^, ^157^, ^158^, ^159^

### 6.2 Site‐specific seeding and colonization

The seeding of infiltrating tumor cells in the PMME and their subsequent colonization and progression in specific organ sites were summarized in several reviews.^7^, ^36^, ^58^, ^154^, ^160^, ^161^, ^162^

As mentioned earlier, DTC that succeeded to complete the migratory journey through the endothelium of “preferred” specific organs need the support of the PMME (and subsequently the MME) in order to survive, proliferate and overcome unfavorable and hostile microenvironmental factors.

Below are several examples of site‐specific colonization of DTC. Neurogenesis, angiogenesis and various growth factors take part in the poststroke tissue repair process, which leads to the regeneration of new blood vessels and new neurons. In a recent study, we demonstrated that melanoma cells utilized (hijacked) factors in the poststroke regenerative neurovascular niche to form brain metastasis. Specifically, we demonstrated that brain microenvironmental cells such as ECs or astrocytes from the peri‐infarct region of the brain or cells that were subjected in vitro to stroke‐inducting conditions (oxygen and glucose deprivation) secreted factors that promoted brain metastasis formation by melanoma cells.^163^

VCAM‐1 expressed by disseminating breast cancer cells binds to α4‐integrin expressed by metastasis‐associated macrophages. This binding activates Akt signaling in lung‐metastasizing cancer cells, thereby protecting them from apoptosis.^164^

Propagation of invading cancer cells can be promoted by both tumor‐derived factors and those emitted from the PMME/MME. For example, extracellular vesicles secreted from highly metastatic clones of a human osteosarcoma are capable of inducing a high metastasizing behavior on poorly metastatic clones from the same tumor.^165^

The mechanisms by which immunity is involved in resistance against tumor and metastasis formation, propagation and progression are, in most cases, uniform for most cancer types.^166^, ^167^, ^168^ Moreover, immunotherapy harboring great promise for cancer patients targets a variety of mechanisms that operate in most cancer types. Reports on specific involvement of tumor immunity or of lymphoid/myeloid cells, or their products, in site‐specific metastasis are rare with some exceptions.

Infiltrating myeloid‐derived cells promoted the formation of breast cancer metastases in the liver but did not affect the formation of lung and bone metastasis. Depleting the granulocytic component of the infiltrate resulted in significantly impaired formation of liver metastases. Neutrophils that infiltrated the liver metastases expressed the malignancy enhancing N2 phenotype.^169^

CXCL12 and TRAIL expressed in the MME of the boneregulate Akt signaling and survival responses in bone‐metastasizing breast cancer cells,^170^ and neutrophil‐derived leukotrienes supported the colonization of lung‐metastasizing breast cancer cells.^171^

IL‐1β‐expressing prostate cancer cells generated a higher load of bone metastasis than prostate cancer cells that do not express this cytokine. Moreover, IL‐1β‐expressing cells created an MME that supported bone metastasis formation also by prostate cancer cells that do not express IL‐1β. This suggested that IL‐1β may promote bone colonization by prostate cancer.^172^

The fractalkine axis (CX_3_CL1‐CX_3_CR1) plays an important regulatory role in organ‐specific peritoneal colonization of ovarian cancer. A high expression of CX_3_CR1 by the tumor cells correlates with a highly malignant phenotype while its downregulation reduces its interaction with CX3CL1 expressed by human mesothelium and reduces metastasis.^173^

## 7 DORMANCY OF DISSEMINATING TUMOR CELLS AT SITE‐SPECIFIC MMEs

Upon arrival at the specific metastatic site, DTC either proliferate and progress toward full blown, clinically detectable metastasis or remain dormant. Tumor dormancy is manifested either as quiescent solitary cells or small clusters of micrometastases (cellular dormancy), or as single cells or cell clusters that do not grow due to an equal rate of proliferation and cell death (mass dormancy)^174^ (Figure 1B).

Dormant micrometastatic tumor cells reside in various MMEs alongside with frank metastases.^175^, ^176^, ^177^

Several studies indicated that the specific organ MME dictates dormancy of DTC and that dormancy functions as a survival strategy of such cells when their progression toward frank metastasis is blocked due to a variety of reasons.^178^, ^179^, ^180^ MME‐derived signals such as antitumor immune responses^180^ induce tumor dormancy by restraining the proliferation of micrometastatic cells, thereby blocking their progression toward metastasis.

Dormancy induction of bone‐metastasizing prostate cancer cells represents a typical case of MME‐regulated dormancy at a specific organ site. TGFβ2,^181^ bone morphogenetic proteins (BMP)^182^ and other factors^183^ induce dormancy of prostate cancer cells in the bone. The mechanism underlying this site‐specific dormancy is an alteration in the ERK and p38 MAPK signaling ratio so as to create a high p38 and a low ERK signaling.^184^ A similar mechanism was indicated in the induction of dormancy in lung‐metastasizing neuroblastoma cells.^185^

The concept of “Microenvironmental Control” was independently proposed by Klein and Klein^186^ and by Bissell and Hines.^187^ According to this concept, cellular or soluble host‐derived factors derived from outside the immunity system may kill cancer cells or cause their growth arrest.^187^, ^188^ The microenvironmental control concept was extended by our group to include innate “moonlighting” molecules that function also as organ‐specific resistance factors that keep dormant DTC in check by restraining their proliferation and progression.^185^ The beta‐subunit of human hemoglobin (HBB) produced by alveolar epithelial and ECs mediated growth arrest and apoptosis of lung‐metastasizing neuroblastoma macro‐ as well as micrometastasis. A HBB‐derived peptide inhibited xenografts of human neuroblastoma tumors as well as spontaneous lung and bone marrow metastases in nude mice.^189^ Preliminary unpublished results indicate the presence of melanoma‐restraining molecules in mouse brain. These results suggest the existence of innate nonimmunological organ‐specific antitumor‐resistant mechanisms.

The MME plays an important role also in mechanisms that awaken dormant micrometastatic tumor cells.^190^ Some MME‐derived signals that awaken dormant tumor cells have been identified. For example, it was suggested that bone marrow‐derived granulin is one of such signals.^191^ Inhibition of the TGF‐β signaling cascade or upregulation of p38 awakened dormant tumor cells^192^ and stromal inflammation reactivates dormant breast cancer cells.^193^

## 8 DIFFERENTIAL RESPONSES OF METASTATIC CELLS TO THERAPY IN DIVERSE MMEs

Primary and metastatic tumor cells and their microenvironments control each other's phenotype.^19^, ^20^, ^29^ This includes response to antitumor therapy. For example, in lung cancer patients, brain metastases responded less well than primary tumor cells to immune checkpoint inhibition. This result was possibly due to a diminished infiltration of PD‐1‐positive T cells to the brain metastatic lesions.^194^

Do metastatic tumor cells residing in a particular MME respond to anticancer therapy similarly or differently than tumor cells (originating in the same ancestral primary tumor) residing in a different MME? This question is obviously of high clinical relevance.

A meta‐analysis of response of breast cancer metastasis to various treatment modalities in relation to metastatic sites revealed that HR‐positive metastases located in different organs and subjected to certain forms of targeted therapy responded similarly. On the other hand, hormonal therapy as well as HER2‐targeted therapy were more effective in visceral metastases than in nonvisceral metastases.^195^

Several studies that dealt with the response of metastasis in different MMEs concluded that metastasis lodging in diverse environments exhibit a differential responsiveness to cancer therapy.^196^, ^197^, ^198^, ^199^ For example, colorectal cancer‐derived lung and brain metastases had higher KRAS mutation rates than other metastatic sites. This may explain their poor response to anti‐EGFR therapy.^170^

In breast cancer, RANKL and Jagged1‐targeting molecules as well as EGFR inhibitors reduce bone metastasis. Antibodies directed against the astrocyte‐elevated gene‐1 protein (encoded by the MTDH gene) diminish lung metastasis and various anti HER2 agents delay brain metastasis.^200^

In order to improve the efficiency and success of both current and future treatment modalities of metastatic diseases, the issue of differential responses of metastatic cells inhabiting different MMEs should be thoroughly investigated. Precision medicine should include the introduction of tailor‐made therapies for cancer cells that metastasize to distinct MMEs.

## 9 BRAIN METASTASIZING TUMOR CELLS COLONIZE A UNIQUE MICROENVIRONMENT

“The brain is a complex biological organ of great computational capability that constructs our sensory experiences, regulates our thoughts and emotions, and control our actions”.

Eric Kandel

The brain differs from other organs in many parameters including but not limited to a unique anatomy, cellular heterogeneity and molecular milieu. All these factors create a supercomplex network of signaling pathways.

Metastasis to the brain constitutes a major unmet clinical challenge.^201^, ^202^, ^203^ Between 20% and 40% of cancer patients will develop brain metastasis. Patients with lung, breast, colorectal and renal cancer as well as with melanoma are most prone to develop brain metastasis. Their prognosis remains poor.^204^, ^205^

**FIGURE 2:**
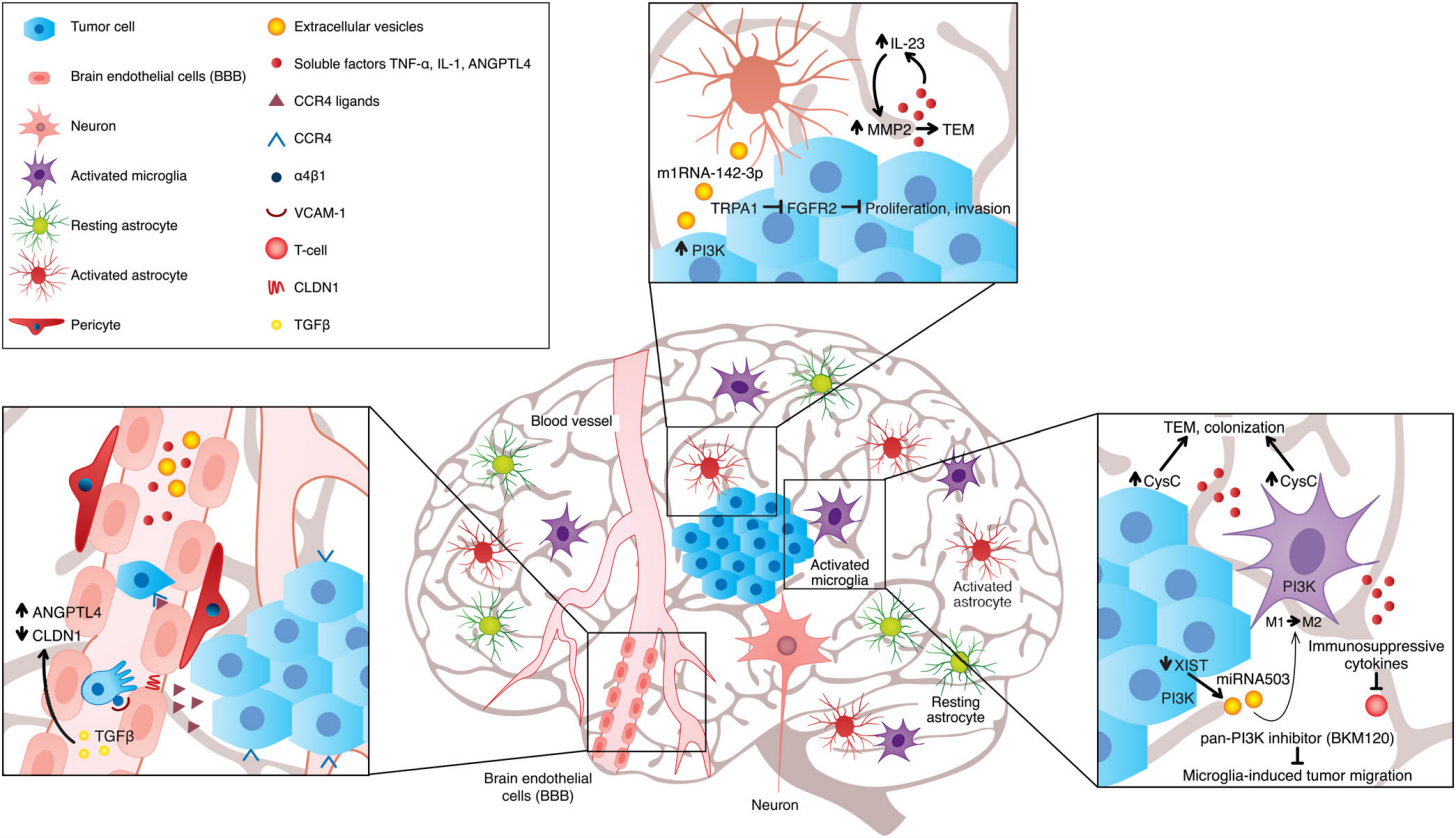
Brain‐specific metastasis and the brain microenvironment. Brain metastasizing cells interact with brain microenvironmental cells such as endothelial cells,^80^, ^129^, ^130^, ^217^, ^218^, ^231^, ^232^ astrocytes^238^, ^243^ and microglia^250^, ^253^, ^255^ and with other components of the brain microenvironment (eg, ECM). These interactions may either inhibit or promote brain metastasis

In addition to metastasis genes that determine the formation of brain metastasis,^127^, ^206^ the establishment of brain metastasis, similarly to metastasis formation in other organs, largely depends on multiple and varied interactions of brain metastasizing cancer cells with cellular and molecular components of the brain^2^, ^207^ (Figure 2).

It is important to note that many of such interactions take place simultaneously and may be affected by factors from compartments external to the brain. This calls for caution in the interpretation of results derived from analyzing tumor‐brain cross talk. Here, we summarize several instances of tumor‐brain cross talk and their biologic consequences. We will include studies from our lab focusing on melanoma brain metastasis. Neurogenesis plays an important role in metastasis formation of several tumors (see later) and obviously also in brain metastasis.^208^ Neurotrophins, cytokines that regulate neurogenesis, facilitate trans BBB invasion of brain metastasizing melanoma cells by promoting the synthesis of ECM and endothelial basement membrane‐degrading enzymes.^209^

The enhanced infiltration of the melanoma cells through the BBB endorses brain metastasis.

Neural repair after stroke involves neurogenesis among other proliferative programs. A recent report indicated that regenerating neurogenesis induced by stroke promoted brain melanoma metastasis.^163^

The BBB structure composed of ECs, pericytes and astrocytes prevents the nonselective entry of unwanted particles and molecules into the CNS.

### 9.1 Endothelial cells

The specific features and properties of the BBB (and its main component, the ECs of the brain) distinguish it from vasculatures of other organs.^210^, ^211^, ^212^, ^213^, ^214^, ^215^, ^216^

As pointed out above adhesion of tumor cells to endothelium is the first step of transendothelial migration. Below is a summary of several studies that characterized molecules and signaling pathways involved in the adhesion of cancer cells to brain endothelium and in their transendothelial migration to form brain metastasis. An early study revealed that TNF or IL‐1 promoted the adhesion of murine tumor cells to brain endothelium.^217^ VCAM‐1 is a major player in the interaction of cancer cells with its ligand α4β1 integrin expressed by endothelia of different organs including brain. This interaction facilitated the formation of breast cancer brain, lung and bone metastasis.^218^

Tight junctions play an important role in sustaining the barrier function of the brain endothelium.^219^

Results from our lab revealed a set of observations that, at first glance, seemed unrelated. Human melanoma brain metastasis variants^177^ express lower levels of claudin‐1 (CLDN1), a tight junction protein, than local melanoma variants derived from the same patients. Brain but not lung ECs express CLDN1. Expression levels of CLDN1 in clinical samples of benign nevi expressed significantly higher levels of CLDN1 than melanoma metastases. CLDN1 overexpression in brain metastasizing cells increases adhesion but reduces transmigration of melanoma cells through brain but not through lung endothelial monolayers. Furthermore, overexpressing CLDN1 in melanoma cells eliminated the capacity of such cells to form brain but not lung metastasis. The mechanism underlying these observations is that CLDN1‐expressing melanoma cells and CLDN1‐expressing brain ECs form homotypic CLDN1‐CLDN1 interactions, which strongly bind tumor cells to ECs. This binding inhibits transmigration. Such interactions do not occur in the lung where ECs do not express CLDN‐1 so that melanoma cells are not blocked from metastasis formation in the lungs.^129^

The *Rho*‐associated protein kinase (*ROCK*) signaling pathway is involved in the interaction of metastatic melanoma cells with the brain endothelium. The inhibition of the kinase augmented the adhesion of the melanoma cells to endothelium, increased their transendothelial migration and promoted the formation of brain metastasis.^220^

The BBB of brain metastasis is leaky.^221^ The leakiness is mediated, at least in part, by soluble factors secreted from brain metastasizing cancer cells. One of such factors is angiopoietin‐like 4 (ANGPTL4).^222^, ^223^ Possibly related to its capacity to disrupt the integrity of the BBB, we found that ANGPTL4, a marker of human melanoma brain metastasis whose expression is regulated by TGFβ1,^177^ promotes the malignancy of cutaneous melanoma cells and augments their potential to form brain metastasis.^130^

The BBB produces a variety of cytokines and other factors such as extracellular vesicles and responds to factors produced by tumor cells or by other brain microenvironmental cells. Such factors may have a far reaching, however opposing, impact on the capacity to form brain metastasis.^163^, ^224^, ^225^, ^226^, ^227^, ^228^

### 9.2 Pericytes

Although the physiological and some pathological functions of pericytes (including their role in metastasis) have been reviewed,^229^, ^230^ the information regarding the specific role of these cells in brain metastasis is rather limited. Below is a summary of studies showing that pericytes do take part in the regulation of brain metastasis.

Pericytes regulate vascularization of brain metastasis by being the main source of the connective tissue in such metastasis.^231^

The formation of brain metastasis by mouse breast cancer cells is associated with an alteration in the representation of pericyte subpopulations. An increase of desmin‐expressing pericytes and a decrease of CD13‐expressing pericytes in the brain is associated with increased permeability of the barrier separating breast cancer brain metastasis from the circulation.^232^, ^233^

Brain pericytes exert an antimetastatic effect by regulating BBB resistance to the formation of lung cancer brain metastasis. Conditioned medium of pericytes suppressed the proliferation of the cancer cells.^85^

### 9.3 Astrocytes

The complex roles of astrocytes in brain metastasis^234^ revealed that astrocytes may fulfill a dual role (Janus face; Yin‐Yang functions) with respect to brain metastasis.^204^, ^235^, ^236^ However, the number of reports on brain metastasis‐promoting functions of astrocytes exceeds significantly that of reports on astrocyte‐mediated antimetastasis functions.

A study demonstrating that astrocytes exert antibrain metastatic activity showed that astrocytes in breast and lung brain‐metastasis secrete plasmin, which cleaves FasL expressed by astrocytes, thereby generating a death signal for the metastatic cells. Plasmin also inactivated L1CAM expressed by the metastatic cells, which was utilized by them as a spreading factor. The antimetastatic function of astrocyte‐derived plasmin was counteracted by cancer cell‐derived serpin.^237^

Transient receptor potential ankyrin 1 (TRPA1) is an ion channel that drives brain metastasis of lung cancer by activating fibroblast growth factor receptor 2 (FGFR2). Astrocytes block metastasis by downregulating TRPA1 via miRNA‐142‐3p.^238^

Below are some recent reports on the prometastatic functions of astrocytes and on the molecular mechanisms by which these brain cells exert their promoting activities on brain‐metastasizing cancer cells.

Astrocytes promote the development of brain metastasis by upregulating the expression of survival genes and by activating tumor promoting pathways in brain metastasizing cells.^239^, ^240^

Extracellular vesicles from brain‐metastasizing melanoma cells activate proinflammatory signaling pathways in astrocytes.^241^ Such pathways usually support tumor progression toward metastasis. Brain‐metastasizing breast cancer cells express upregulated levels of ID‐2 (inhibitor of differentiation 2). This protein, upregulated by astrocyte‐derived bone morphogenetic protein (BMP7), inhibits differentiation, enhances stemness and promotes propagation of brain metastasis.^242^

Two studies from our lab provided additional proof for the support provided by astrocytes to the establishment of melanoma brain metastasis. Human brain‐metastasizing melanoma cells induced the expression by and secretion of IL‐23 from astrocytes. This cytokine, in turn, reciprocally upregulated the secretion of MMP2 from the metastatic melanoma cells, thereby enhancing their invasiveness.^243^ In another study, we showed that brain microenvironmental cells including astrocytes expressed and released CCL17 and CCL22 two chemokine ligands of the chemokine receptor CCR4, which is expressed by a subpopulation of melanoma cells with brain‐metastasizing capacity. This subpopulation is more highly tumorigenic than melanoma cells lacking CCR4. The chemoattraction of CCR4‐expressing melanoma cells to the corresponding brain‐derived ligands was instrumental in the formation of brain metastasis.^80^

### 9.4 Microglia

“As the immune‐competent cells of the brain, microglia play an increasingly important role in maintaining normal brain function.”^244^ Microglia are macrophage‐like resident cells of the brain^245^ involved in brain metastasis.

Similarly to astrocytes, microglia may both support and restrain brain metastasis.^204^, ^235^, ^246^, ^247^ The decision whether microglia will support or antagonize brain metastasis depends to a large extent on a balance between pro‐ and antimetastasis signals generated by the crosstalk between the tumor cells and microglia.

In a study performed in our lab,^248^ we examined the outcome of the bidirectional cross talk between human melanoma cells and microglia by identifying the molecular and functional reprograming occurring in interaction partners after exposure to soluble factors derived from the interacting partner. Some potentially antimetastatic factors and pathways were stimulated by the melanoma‐microglia cross talk. However, these were apparently overshadowed by pathways and factors that promote further tumor progression resulting in a net increase in the malignancy of the melanoma cells.

As with studies on the effect of astrocytes on the formation and maintenance of brain metastasis, studies reporting antimetastatic effects of microglia are less numerous than those reporting the opposite. Below are some examples of studies on anti‐ or pro‐brain metastasis functions of microglia.

Systemic prophylactic administration of CpG‐C, a TLR9 agonist, reduced brain seeding and colonization of mouse and human lung cancer and of mouse melanoma.^228^

Employing a mouse model system, it was found that activated microglia lysed murine and human tumor cells by release of nitric oxide. Activated microglia did not lyse non tumorigenic control cells.^249^

A review of the cross talk between microglia and melanoma brain metastasis^236^ indicated that microglia support such metastasis by attenuating their own phagocytic capacity, the angiogenesis of the infiltrating melanoma cells and by augmenting secretion of vascularization factors. Furthermore, microglia depletion reduced the artificial brain metastasis of breast cancer cells.

PI3K was identified as a master regulator of metastasis‐promoting microglia. Treating brain‐metastasizing mouse mammary tumor cells with a pan‐PI3K inhibitor suppressed significantly metastasis formation by the tumor cells. These results suggest that PI3K inhibition may be developed into a brain metastasis therapy modality.^250^ The finding that the metastasis‐promoting PI3K‐AKT pathway is activated by PTEN inactivation or by NRAS activation^251^, ^252^ has also implications for therapy modalities. XIST (X‐inactive specific transcript) is a noncoding RNA on the X chromosome. In breast cancer patients, low expression levels of XIST were associated with a high brain metastasis burden. Downregulating expression of XIST in a xenograft tumor model promoted M1‐M2 polarization of microglia, induced immunosuppressive properties in these cells and accelerated brain metastasis.^253^

Simultaneously with the development of B16 melanoma brain “metastasis” (intracerebral inoculation), neighboring microglia became increasingly activated, gradually accumulating in the metastatic lesion and thereby promoting further growth of the lesion. Brain “metastasis” was inhibited by microglia depletion in vivo and microglia‐derived MMP3 seemed to facilitate melanoma cell growth.^254^ An intracerebral inoculation was employed in these experiments. It is highly questionable if the brain tumors developing under such circumstances can be characterized as metastasis.

A study from our lab indicated that the secretion of cystatin C (CysC), an extracellular cysteine protease inhibitor, from brain‐metastasizing melanoma cells as well as from microglia is increased after these two cell types engaged in an in vitro cross talk. CysC enhanced the migration of melanoma cells through an in vitro model of BBB and promoted the formation of melanoma three‐dimensional structures in matrigel. Immunohistochemistry (IHC) demonstrated high expression levels of CysC in the brain of nude mice bearing xenografted human melanoma brain metastasis. These results indicate that CysC promotes melanoma brain metastasis.^255^

## 10 PERSPECTIVE

Scores of published studies identified factors in the tumor microenvironment that either promote or restrain metastasis, the final stop of tumor progression.

Are all these factors or only certain combinations thereof necessary in order to generate metastasis? In other words, are we able to separate the wheat from the chaff and to identify the most relevant factors that generate, sustain or resist metastasis? Are there several redundant, nonmutually exclusive signaling pathways that have the capacity to inhibit or promote metastasis? For example, a road block posed by an anticancer drug neutralizing a prometastasis pathway may be circumvented by another, redundant, prometastasis pathway, thereby allowing uninterrupted tumor progression.

Providing answers to the above questions (and to numerous related ones) might enable the construction of a comprehensive, overall big picture of tumor progression toward metastasis. This is a major and challenging task for the metastasis research community in efforts to develop and apply drugs that will prevent metastasis or cure it.

To meet this goal, we have to abandon, as far as possible, reductionism and to employ approaches used in analysis of big data, in Bioinformatics and in Systems Biology.

## CONFLICT OF INTEREST

The authors declare that they have no conflict of interest.
